# Population Genetic Structure and Demographic History of *Primula fasciculata* in Southwest China

**DOI:** 10.3389/fpls.2020.00986

**Published:** 2020-07-02

**Authors:** Guangpeng Ren, Rubén G. Mateo, Elena Conti, Nicolas Salamin

**Affiliations:** ^1^ State Key Laboratory of Grassland Agro-Ecosystems, School of Life Science, Lanzhou University, Lanzhou, China; ^2^ Department of Computational Biology, Biophore, University of Lausanne, Lausanne, Switzerland; ^3^ Departamento de Biología (Botánica), Universidad Autónoma de Madrid, Madrid, Spain; ^4^ Centro de Investigación en Biodiversidad y Cambio Global (CIBC-UAM), Universidad Autónoma de Madrid, Madrid, Spain; ^5^ Department of Systematic and Evolutionary Botany and Botanic Garden, University of Zurich, Zurich, Switzerland

**Keywords:** demography, genetic structure, Hengduan Mountains, population genomics, Quaternary climatic changes

## Abstract

Understanding the factors that drive the genetic structure of a species and its responses to past climatic changes is an important first step in modern population management. The response to the last glacial maximum (LGM) has been well studied, however, the effect of previous glaciation periods on plant demographic history is still not well studied. Here we investigated the population structure and demographic history of *Primula fasciculata* that widely occurs in the Hengduan Mountains and Qinghai-Tibetan Plateau. We obtained genomic data for 234 samples of the species using restriction site-associated DNA (RAD) sequencing and combined approximate Bayesian computation (ABC) and species distribution modeling (SDM) to evaluate the effects of multiple glaciation periods by testing several population divergence models and demographic scenarios. The analyses of population structure showed that *P. fasciculata* displays a striking population structure with six groups that could be identified genetically. Our ABC modeling suggested that the current groups diverged from ancestral populations located in the eastern Hengduan Mountains after the largest glaciation occurred in the region (~ 0.8–0.5 million years ago), which is consistent with the result of SDMs. Each current group has survived in different glacial refugia during the LGM and experienced expansions and/or bottlenecks since their divergence during or across the following Quaternary glacial cycles. Our study demonstrates the usefulness of population genomics for evaluating the effects of past climatic changes in alpine plant species with shallow population structure.

## Introduction

Plant populations are not randomly arranged assemblages of genotypes but are structured in space and time (Loveless and Hamrick, 1984). Because of the limited mobility of plants, their genetic structure implies spatial structure, where genetic differentiation increases with geographic distance (Wright, 1943). Yet, recent empirical studies have put forward that geographic distance by itself fails to fully explain the genetic variation observed in natural systems (e.g., Shafer and Wolf, 2013). In fact, geographic, environmental, historical, and intrinsic factors (e.g., mating system) have been suggested to simultaneously act as drivers of spatial genetic patterns at different spatial scales (Wang et al., 2013; Muñoz-Pajares et al., 2017; Ren et al., 2017). Identifying the factors that drive the genetic structure of a plant species is an important first step not only to understand speciation, adaptation, and genetic change (Antonovics, 1968), but also to help in population management. In the latter case, the spatio-temporal dynamics of population histories can profoundly impact their future evolutionary potential (e.g., Lanier et al., 2015). This is especially true for climate-sensitive species inhabiting highly fragmented environments, such as mountain ranges.

**Figure 1:**
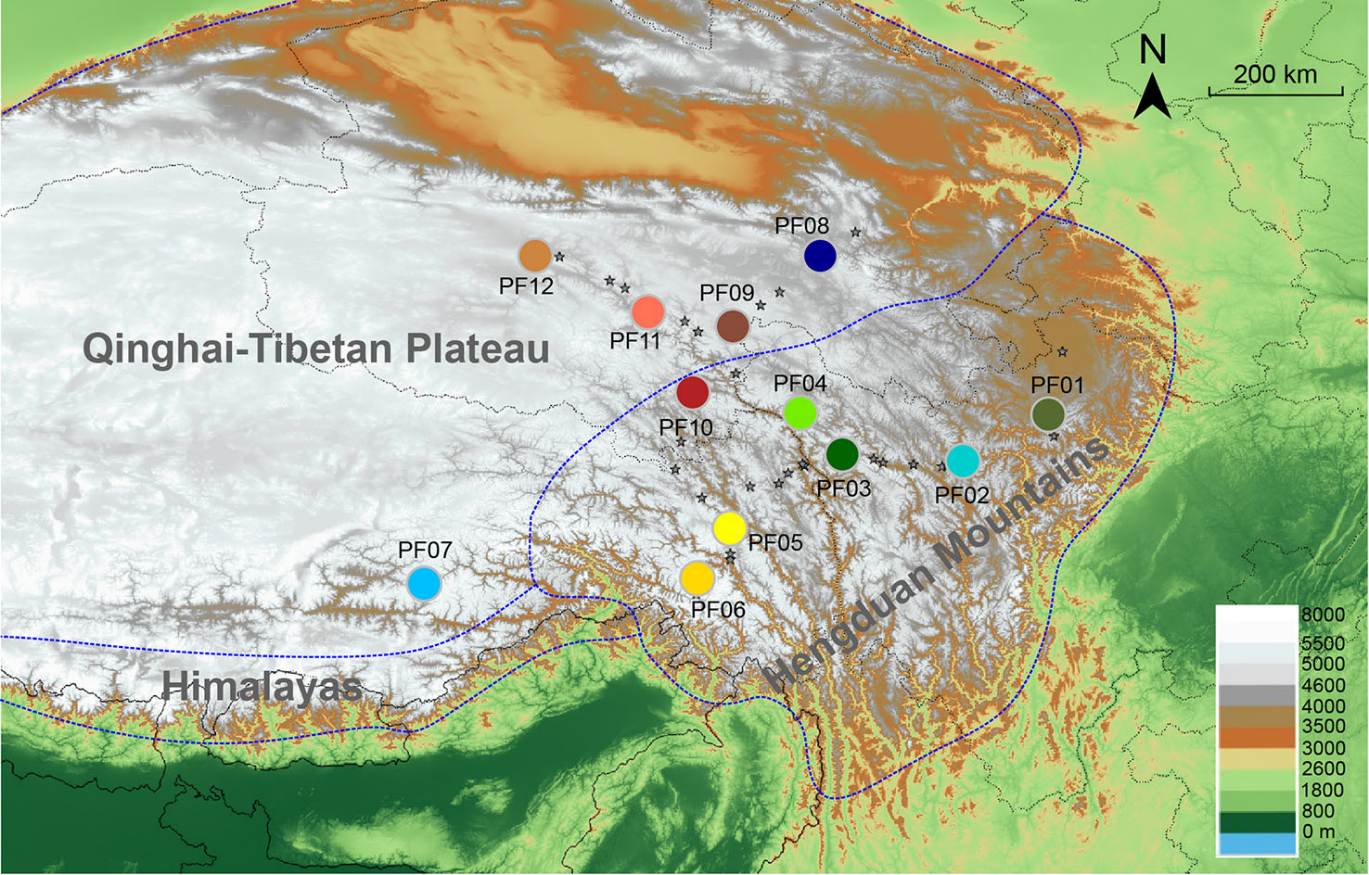
Sampling locations of all 61 populations of *P. fasciculata* (gray stars) and the 12 selected populations (large colored circles) used for genomic analyses in this study. The three regions of the Tibeto-Himalayan region were delineated by blue dotted lines.

One of the key high-altitude biodiversity hotspots in the world where these processes can be studied are Mountains of Southwest China, i.e., the Hengduan Mountains region. They were formed by recent uplifts of mountains during the late Miocene and Pliocene (Li and Fang, 1999; Myers et al., 2000; Zheng et al., 2000; Mulch and Chamberlain, 2006; also reviewed in Favre et al., 2015; Renner, 2016; Muellner-Riehl, 2019). Additionally, the climate in the region and the Qinghai-Tibetan Plateau (QTP) has changed drastically during the Quaternary, and the extent and timing of glaciations remain controversial, especially for the old glaciations (Ou et al., 2015; Muellner-Riehl, 2019). However, recent studies have suggested that four major glaciations have likely occurred since 1.2 million years ago (Ma), which are the Xixiabangma (0.8–1.17 Ma), Naynayxungla (0.5–0.72 Ma), Guxiang (0.13–0.3) and the Last Glaciation (0.01–0.07 Ma; Shi, 2002; Zheng et al., 2002; Ou et al., 2015). The Naynayxungla Glaciation is thought to be the largest glaciation that occurred in this region (Shi, 2002; Zheng et al., 2002; Ou et al., 2015). The origin and maintenance of the high biodiversity in this region are the result of its specific topographic features and profound ecological heterogeneity created by the historical orogenesis and associated climatic changes (Wu, 1987). Today, the Hengduan Mountains are characterized by parallel and deep North-South oriented valleys surrounded by high mountain peaks (**Figure 1**). The mountains display drastic altitudinal variations ranging from 1,000 m to numerous peaks above 6,000 m, and the area is particularly vulnerable to climate change (Zheng, 1996; Yao et al., 2007). With such a complex geological, climatic, and ecological diversity, the region has attracted attention of numerous biologists to study the factors affecting species diversification and evolution (e.g., Xing and Ree, 2017; reviewed in Qiu et al., 2011; Liu et al., 2014; Wen et al., 2014; Mosbrugger et al., 2018). Some studies focused on species-level diversification that resulted from the uplift of the QTP and Hengduan Mountains (e.g., Liu et al., 2002; Liu et al., 2006; Ren et al., 2015), while others looked at intraspecific divergence to investigate the effects of past geological events and Quaternary climatic fluctuations on population genetic structure (e.g., Wang et al., 2009; Liu et al., 2013). For example, studies on *Quercus aquifolioides* (Du et al., 2017), on several closely related *Picea* species (Li et al., 2013)and on *Taxus wallichiana* (Liu et al., 2013) have suggested that plant species in the Hengduan Mountains tend to show long-term demographic stability and survival in multiple refugia. Although numerous phylogeographic studies on herbs, shrubs, and trees in this region have suggested several phylogeographic patterns (summarized in Muellner-Riehl, 2019), a comprehensive understanding of the factors triggering current genetic structure and a detailed demographic scenario in response to the Quaternary climatic fluctuations in this region are still unclear because of the limited genetic information used in most previous studies.

Integrative approaches combining population genomics (e.g., Ren et al., 2017) with niche modeling (Guisan and Zimmermann, 2000) have helped to understand current spatial genetic patterns and the processes (Theodoridis et al., 2016). Population genomic data can provide accurate estimates of genetic structure (Avise, 2010; Narum et al., 2013) and increased accuracy when estimating demographic parameters (e.g., Emerson et al., 2010; Bourret et al., 2013; Lanier et al., 2015; Ren et al., 2017), whereas species distribution models allow to predict geographic areas that are part of the ecological niche of species at different temporal and spatial scales. A recent study based on these approaches has significantly advanced our understanding of the response of alpine plant species to Quaternary climatic changes in the Himalayas (Ren et al., 2017). Although Next-Generation Sequencing (NGS) methods have recently become cost-effective, the application of population genomics on the taxa distributed in the Hengduan Mountains remains rare because of its remoteness and inaccessibility, and consequently, such genomic level studies are particularly needed for this region to provide a better understanding of evolutionary history of species.

Here we focus on *Primula fasciculata* (Primulaceae), one of the most widely distributed alpine plant species in the Hengduan Mountains and QTP (Hu and Kelso, 1996; **Figure 1**). It is an insect-pollinated, heterostylous, herbaceous, perennial plant that occurs in diverse habitats at elevations ranging from 2,900 to 5,000 m. As an outcrossing small herb of variable height (2–10 cm), *P. fasciculata* disperses its seeds largely by gravity and usually grows in wet meadows or along hill-streams (Hu and Kelso, 1996; Richards, 2003). A recent study, focused on *P. fasciculata* and its two closely related species (i.e., *P. tibetica* and *P. nutans*) at species level and investigated interspecific divergence and the factors that affected the maintenance of species boundaries, has indicated that *P. fasciculata* diverged from *P. tibetica* during the Pliocene and experienced expansion during the Quaternary (Ren et al., 2018). However, the population structure and demographic history of *P. fasciculata* and the factors affecting its demography were not involved in Ren et al. (2018). Here, we use an integrative approach combining genomic phylogeography with niche modeling to test several demographic scenarios corresponding to specific hypotheses related to the effects of Quaternary climatic fluctuations on alpine species in this region. The aims of our study are to: i) identify the population structure of *P. fasciculata* and understand the factors driving it; and ii) compare several detailed demographic scenarios for *P. fasciculata* using ABC modeling and test these hypotheses with species distribution models to evaluate the effects of Quaternary climatic changes on the evolution of this species.

## Materials and Methods

### Data Set

The data set of *P. fasciculata* (Dryad doi: https://doi.org/10.5061/dryad.tt8n46q; File 1-D2) used in Ren et al. (2018) that comprises 234 individuals from 12 populations with 17.8% missing data was re-analyzed in this study to further investigate the population structure and demographic history of this species. In order to obtain estimates of neutral population genetic structure, the detected 106 outlier SNPs by both BAYESCAN and LOSITAN in this species (Ren et al., 2018) were removed for the downstream analyses. Therefore, all results in this study were derived from the neutral data set containing 5,980 single-SNP loci. This neutral data set in Ren et al. (2018) was used only to test whether population divergence of the neutral genomic fractions was driven by geographical or environmental or both factors. The result showed that both factors played a role, which was the only result derived from this data set in our previous paper. In this study, on the basis of this data set, we did multiple population structure analyses and ABC modeling to investigate population structure and demographic history of this species. Therefore, the hypotheses behind and the analytical methods were completely different between the two studies.

The 12 populations were selected from our 54 sampled populations to be representative of both the geographical distribution and the diversity of ecological niches of this species. We estimated the latter by extracting the 19 bioclimatic variables of WorldClim (http://www.worldclim.org/current) from the occurrences of the individuals sampled in the 54 populations. We did a principal component analysis (PCA) using the *prcomp* function in the *stats* package of R and identified the 12 populations based on the PC1 and PC2 axes (explained nearly 82% of the variance; **Figure S1**). Seventeen to 20 individuals were sampled in each population, making sure that all individuals sampled were at least 20 m apart. The detailed location information of the 12 populations was listed in Supporting Information **Table S1**.

### Characterization of Population Genetic Structure

Population genetic structure of *P. fasciculata* was estimated by using the Bayesian method implemented in STRUCTURE 2.3.4 (Pritchard et al., 2000) and by principal components analysis (PCA). Structure analyses were performed under the “Admixture model” and the “Correlated allele frequency model” with K-values ranging from 1 to 12. Ten independent runs were performed for each value of K using 1 × 10^5^ generations for the burnin and 2 × 10^5^ generations for the sampling. The optimal K was chosen using the delta-K method of Evanno et al. (2005) as implemented in STRUCTURE HARVESTER (Earl and VonHoldt, 2012). The coefficient for cluster membership of each individual was averaged across the ten independent runs using CLUMPP (Jakobsson and Rosenberg, 2007) and plotted using DISTRUCT (Rosenberg, 2004). PCA was performed with the *glPCA* function in *adegenet* package (Jombart, 2008) in R to identify the major axes of variation of the populations.

Pairwise *F*_ST_ values and analysis of molecular variance (AMOVA) among populations were calculated in GENODIVE v.2.0b27 (Meirmans and Van Tienderen, 2004), and significance was determined using 1 × 10^4^ permutations. AMOVA for populations that were further clustered into several groups based on the STRUCTURE and PCA results (**Table S2**) was applied to evaluate which grouping strategy explains the highest percentage of total variance among groups, which was used to select the most likely strategy of the grouping for ABC modeling.

The first three components of the PCA performed on the genetic data and the geographic coordinates (latitude and longitude) of the 12 populations were used in a Procrustes analysis using the R package *vegan* (Oksanen et al., 2013). This analysis minimizes the sum of squared Euclidean distances between two sets of points by rotating one set of points to match the other, while preserving the relative distances among all points within the map (Wang et al., 2012). The similarity of the two maps is quantified using the Procrustes similarity statistic *t*_0_ (Wang et al., 2010; Wang et al., 2012). We used the *protest* function in *vegan* to test the probability of observing a similarity statistic higher than the observed to if no geographic pattern is assumed using 1 × 10^5^ permutations (Wang et al., 2012).

We further used BARRIER v2.2 (Manni et al., 2004) to compute the Monmonier's maximum-difference algorithm for identifying biogeographic boundaries or areas exhibiting the largest genetic discontinuities between population pairs based on pairwise genetic distances (*F*_ST_). We randomly selected 5,000 loci from the neutral data set 100 times to generate 100 *F*_ST_ distance matrices by using *populations* module in the STACTS v1.30 (Catchen et al., 2013). The number of barriers was set to vary from 1 to 10, reflecting their descending order of relative importance (“priority”) for genetic dispersion (Manni et al., 2004). The robustness of the genetic boundaries was assessed by running BARRIER on the 100 *F*_ST_ distance matrices.

### Estimates of Historical Demography

**Figure 2:**
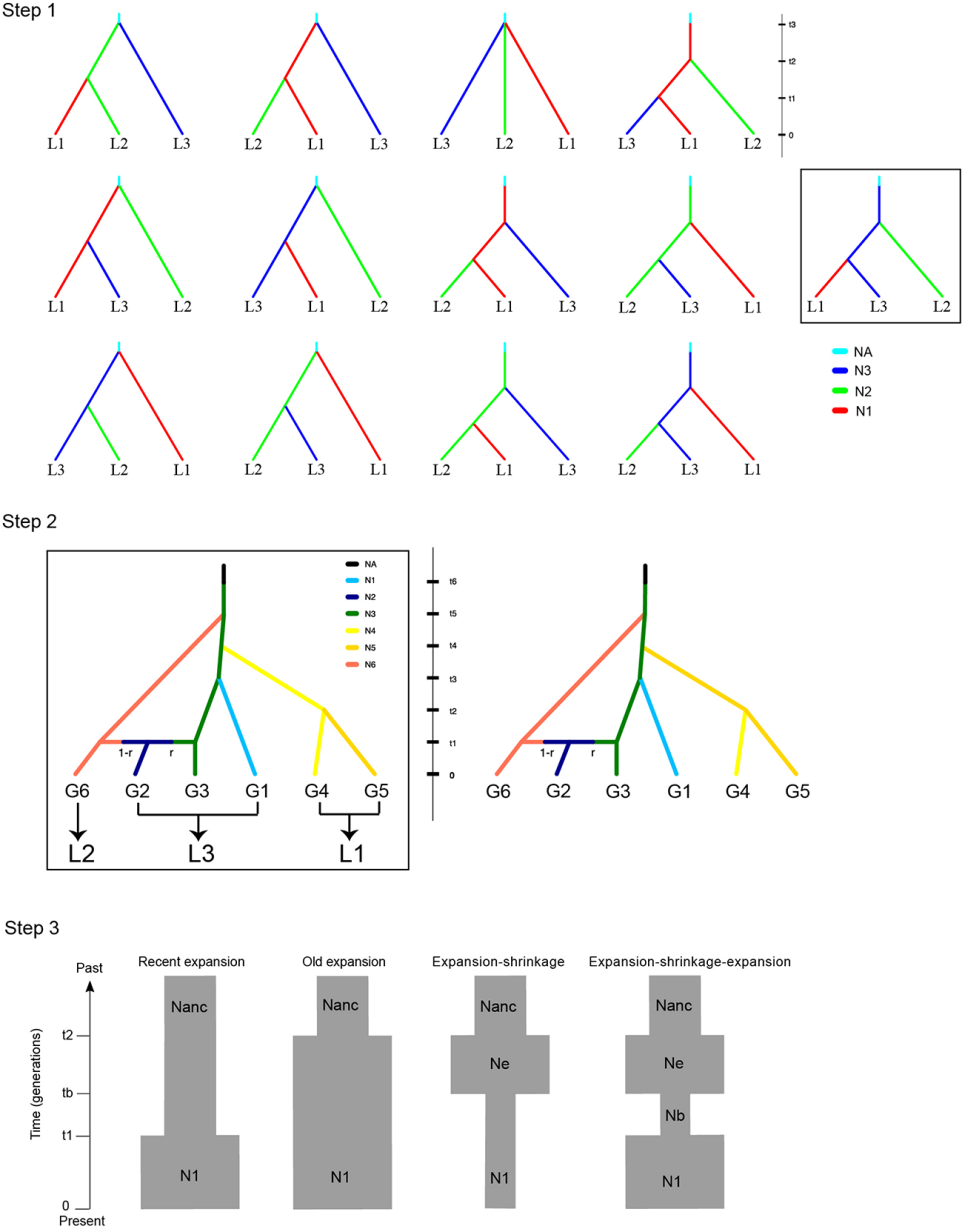
Alternative demographic scenarios for the three steps analyzed by DIY-ABC. The best-fit scenario was indicated by square in steps 1 and 2.

To decipher the historical demography of *P. fasciculata*, we estimated divergence times, admixture, and changes in population sizes among different groups of population using approximate Bayesian computation (ABC) modeling. We stratified the procedure in three steps (**Figure 2**): (1) we investigated the most likely tree topologies for the three main lineages (see *Results*) that were identified by the PCA analyses among 13 scenarios describing all possible topologies (**Figure 2**; **Table S2**); (2) we split the three main lineages into six groups based on AMOVA analysis on multiple grouping strategies (see more details in *Results*), i.e., L3 was split into G3, G1, and G2; L1 was split into G4 and G5; L2 was not split and renamed as G6 (see *Results*; **Figure 2**; **Table S2**). Based on the best-supported tree topology obtained in (1) and the STRUCTURE result, and given the fact that G1 had the highest altitude distribution (**Table S1**) and was sampled in the westernmost of the distribution of the species (suggesting that G1 was not likely ancestral), we fixed the ancestor of G3 as the most recent common ancestor for all groups, then G6 and G1 originated from G3, and G2 originated from the admixture between G3 and G6. Finally, we set two scenarios to model the divergence order of G4 and G5 from G3. This step was conducted to estimate the divergence times among the six groups; (3) we tested changes in population sizes of each of the six groups in the recent past among four scenarios (**Figure 2**; Ren et al., 2017): i) old expansion; ii) recent expansion; iii) expansion followed by shrinkage; iv) expansion followed by shrinkage and a new expansion event. Five individuals that had the least missing data from each of the 12 populations were selected for steps 1 and 2 to reduce computational time. For step 3, we used those same five individuals for the two groups (i.e., G3 and G6) that contained multiple populations, whereas all individuals were used for the four groups (i.e., G1, G2, G4, and G5) that included only one population.

For each step, we tested different scenarios using DIY-ABC v.2.1.0 (Cornuet et al., 2010; Cornuet et al., 2014). We selected for these analyses a single SNP per locus, which had to be present in (i) at least 80% of the individuals from each lineage/group and (ii) all lineages/groups. We chose a minor allele frequency (MAF) of 0.01 to increase the mean level of genetic variation of both the observed and simulated data sets and to reduce the proportion of loci that may correspond to sequencing errors. The data sets used for the ABC modeling and the distributions of prior probabilities are summarized in **Table S3**. We selected all summary statistics to generate a reference table (on average 10^6^ data sets per scenario). The parameters defining each scenario (i.e., population sizes, divergence times, and/or times of population size changes) were considered as random variables drawn from prior distributions. For each simulation, DIY-ABC drew a value for each parameter from its prior distribution and performed coalescent simulations to generate a simulated pseudo-observed data set (POD) with the same number of gene copies and loci per lineage/group as those observed. It then calculated summary statistic for each POD and the observed data. Based on a distance and a tolerance, it decided for each POD whether its summary statistic was sufficiently close to that of the observed data. We used 1% of the simulated data sets closest to the observed data to estimate the relative posterior probabilities for each scenario *via* logistic regression. Posterior distributions of historical demographic parameters based on the most likely scenario (Cornuet et al., 2010) were estimated. The time parameters are estimated in generations and converted into years by multiplying by the generation time, which was set to one year (Ren et al., 2017). Finally, for each step, we performed an evaluation of the fit of each scenario to the data sets by running a model-checking analysis following Cornuet et al. (2010).

### Species Distribution Models

We generated species distribution models (SDMs; Guisan and Zimmermann, 2000) to evaluate the potential effects of past climatic changes on the distribution of *P. fasciculata* and to compare such effects with the demographic changes modeled by ABC. An ensemble model (Araújo and New, 2007) was generated by the combination of three different statistical techniques: generalized linear model, gradient boosting machine and random forests, as implemented in the R package *biomod2* (Thuiller et al., 2009). A total of 74 occurrences were used as presences data to calibrate the models. The performance of the model was assessed by randomly splitting ten times (cross-validation) the data into an 80% data set to generate the models and a 20% data set to estimate their predictive accuracy (AUC statistic). According to Ren et al. (2017), the paleo-climatic conditions of the last interglacial (LIG) in this area predicted large differences of precipitation compared with the present and the last Maximum Glacial (LGM), resulting in the failure of projection for the LIG, which was also true in the present projection for the LIG (**Figure S2**). Therefore, in this study, we estimated the potential distributions for the 1) the present; 2) the LGM (0.022 Ma), and 3) the Marine isotope stage 19 (MIS19, ~0.787 Ma). As predictors, we used the bioclimatic variables from PaleoClim (http://www.paleoclim.org/, Fordham et al., 2017; Brown et al., 2018; Karger et al., 2017 REF) at 2.5 arcminutes resolution. In order to avoid multicollinearity, we ran a Pearson correlation analysis by pairs for the 14 bioclimatic variables available for all the periods (for MIS19, monthly maximum and minimum temperatures are not available). In each pair with a correlation value greater than 0.7 (Dormann et al., 2013) we removed one climate variable. The climatic variables finally used to calibrate the SDMs were: temperature seasonality (bio4), mean temperature of coldest quarter (bio11), precipitation of wettest month (bio13), precipitation of driest month (bio14), precipitation seasonality (bio15) and precipitation of coldest quarter (bio19).

## Results

### Structuring of Population Genetic Variation

**Figure 3:**
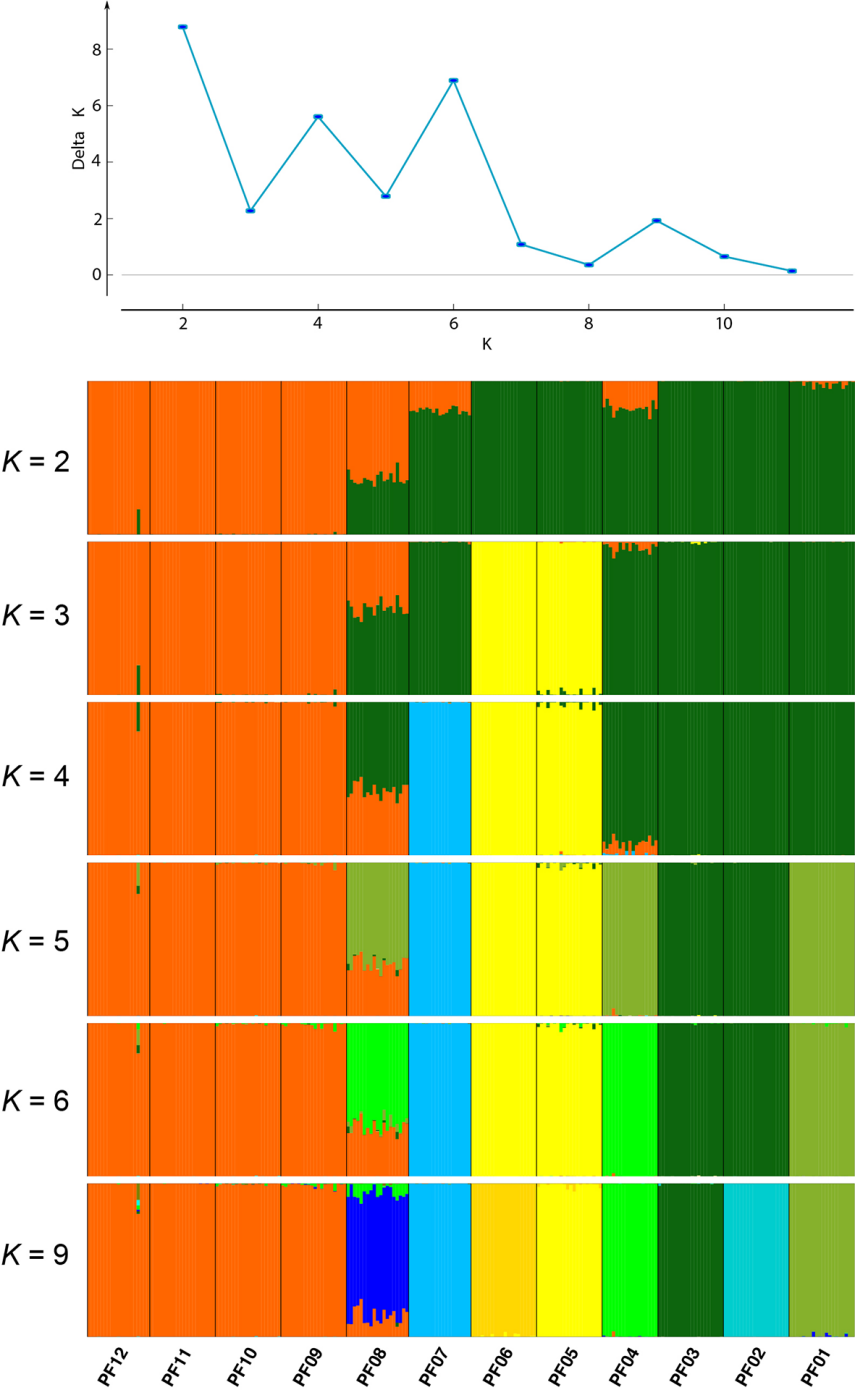
ΔK values identified using STRUCTURE HARVESTER and plots of posterior probabilities for individuals of *P. fasciculata* assigned to *K* genetic clusters from STRUCTURE analyses for *K* = 2–6 and 9. Populations are delimited by black lines, with the corresponding population names listed along the bottom of the plot.

Although the optimal *K* value of the STRUCTURE analyses based on the Δ*K* method of Evanno was *K* = 2, the differences of Δ*K* among *K* values were very small (**Figure 3**). The Δ*K* of the second most probable *K* value (*K* = 6) differed from the optimal one by only 2. Other *K* values (3, 4, 5, and 9) also received considerable support. We decided to show all these *K* values in **Figure 3** and combined them with the PCA results to capture the most reasonable set of lineages for the ABC modeling.

**Figure 4:**
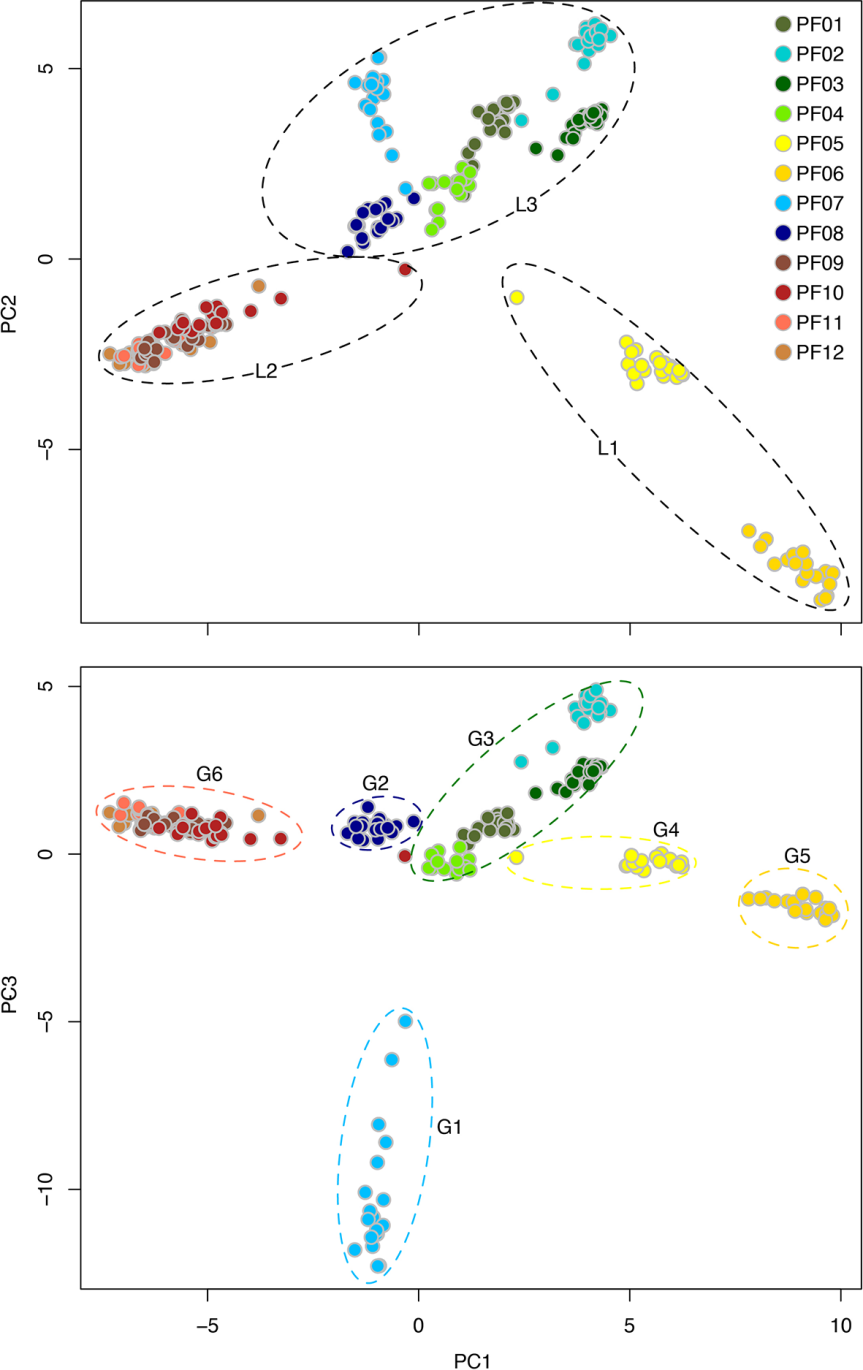
Distribution of individuals of *P. fasciculata* along PC scores (PC1, 13.04% vs. PC2, 7.76%; PC1 vs. PC3, 6.12%) of genetic variation based on the analysis of SNP dataset; individuals are color-coded according to their population identities (see **Figure 1**). The three lineages and six groups used for ABC modeling are indicated.

The first two PCA axes identified three main genetic lineages and explained 13.04% and 7.76% of the total variation, respectively (**Figure 4**). The two southwestern populations (PF05, PF06) and four northwestern populations (PF09-PF12) formed two separate lineages (L1 and L2), while the rest of the populations form a third lineage (L3; **Table S2**). The three main lineages (L1-L3) were used in step 1 of the ABC modeling to identify the most likely population tree topology.

In order to investigate the most likely strategy of grouping for the ABC modeling, we further assigned the 12 populations into four, five, six or seven groups based on the PCA and STRUCTURE analyses (**Figures 3** and **4**). The detailed information of different grouping strategies was summarized in **Table S2**. The strongest signature of population spatial differentiation was obtained by AMOVA analysis (22.2% of total variance; **Table S4**) when populations were assigned to six groups. We therefore used the six groups in step 2 of the ABC modeling to estimate the divergence times among these groups, and in step 3 to estimate the demographic changes for each of them. The six groups were identified as following: the third axis of the PCA (PC3; 6.12% of the total variation) showed a separation of population PF07 from lineage L3, which was also shown when *K* = 4 in the STRUCTURE. We identified this population as G1. Looking at *K* values from *K* = 2 to *K* = 6, population PF08 was always represented as an admixed population, which was labeled as G2. The remaining populations of L3 were grouped as G3. The two populations (L1) that diverged from each other in the PCA (**Figure 4**) were identified as G4 and G5. The four northwestern populations form the sixth group (G6; **Table S2**) evident both in STRUCTURE and PCA.

**Figure 5:**
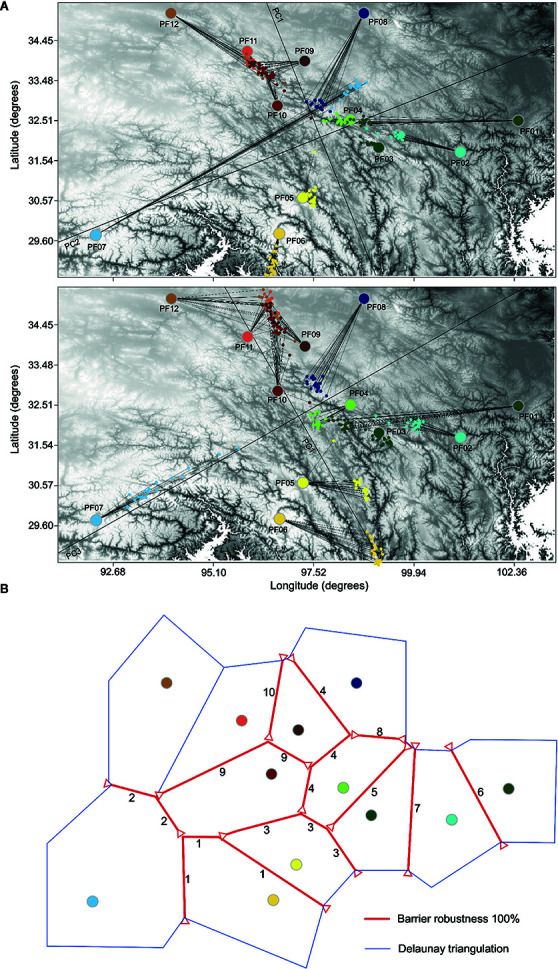
**(A)** Procrustes-transformed PCA plot of genetic variation with each individual of *P. fasciculata* mapped in PC space (the small circles) relative to the geographic location of populations (the larger circles). Black lines show the orientation of the genetic space relative to the geographic longitude and latitudinal axes. The length of the line connecting individuals in PC space to their geographic location represents the extent of the deviation from the expected pattern of genetic variation based on geography. **(B)** Result of BARRIER analysis showing the spatial separation of *P. fasciculata* populations. All the ten barriers (red lines) are highly supported over 100 *F*_ST_ distance matrixes. Barriers are delimited by small red triangle. Numbers (1–10) represent descending order of relative importance (“priority”).

Procrustes analysis was used to quantify the association between the genetic variation of populations and their geographic locations. The first two PC spaces identified a significant similarity score (*t*_0_ = 0.579, *P*_value < 10^−5^), which increased to *t*_0_ = 0.777 when genetic variation in PC1 and PC3 spaces were considered (**Figure 5A**). This was caused by the clear separation of the most geographically isolated population PF07 from other populations by the PC3 axis. Individuals from G6 are genetically more similar with each other than would be expected given the geographic distance among the populations forming this group. The general pattern of association with geography for the rest of populations was robust, indicating high level of population divergence. Such level of divergence was also evident in the BARRIER analysis that gave high support to all ten barriers (bootstrap support 100%; **Figure 5B**). The presence of such strong barriers between *P. fasciculata* populations indicates an abrupt change in the genetic profile of populations across the species distribution. Although the ranking of the population barriers (**Figure S3**) was not in agreement with the STRUCTURE and PCA analyses, the general pattern of spatial genetic structure identified by the BARRIER analysis was consistent with the other analyses. Differentiation among populations was significant, with *F*_ST_ values ranging from 0.088 to 0.604 with a mean value of 0.377 (**Table S5**), which was consistent with AMOVA for the total data set (*F*_ST_ = 0.306; **Table S4**).

### Estimates of Historical Demography

We used a three-step procedure to estimate the demographic history of *P. fasciculata*. Among the 13 scenarios tested in step 1, the scenario depicting an origin of both L1 and L2 from L3, provided the best fit to our data, with posterior probabilities significantly higher than the other scenarios (0.995, 95% credible interval (CI) 0.99, 1.00; **Table S6**; **Figures 2** and **S4**). According to the main tree topology inferred from step 1, the analyses done in step 2 showed that groups G1, G6, and G4/G5 (i.e., alternative scenarios; **Figures 2** and **S4**) originated from G3, while G2 was formed by admixture between G3 and G6. The scenario where G4 originated from G3 and later G5 diverged from G4 fitted the data much better (0.93, CI: 0.93–0.94; **Table S6**; **Figure 2**). A check of the goodness-of-fit of the distributions of the parameters for the scenarios with the real data set further indicated that scenarios 13 and 1 were the best-supported scenarios for step 1 and step 2, respectively (**Table S7**). Modeling the changes in population size for each group (step 3) recovered complicated demographic histories for the six groups. Analyses for G3 supported a scenario of “expansion**–**shrinkage,” while G2, G4 and G6 were better modeled by a scenario of “expansion**–**shrinkage**–**expansion.” The other two groups (G1 and G5) were better modeled by a scenario of “recent expansion” (**Table S6**).

**Figure 6:**
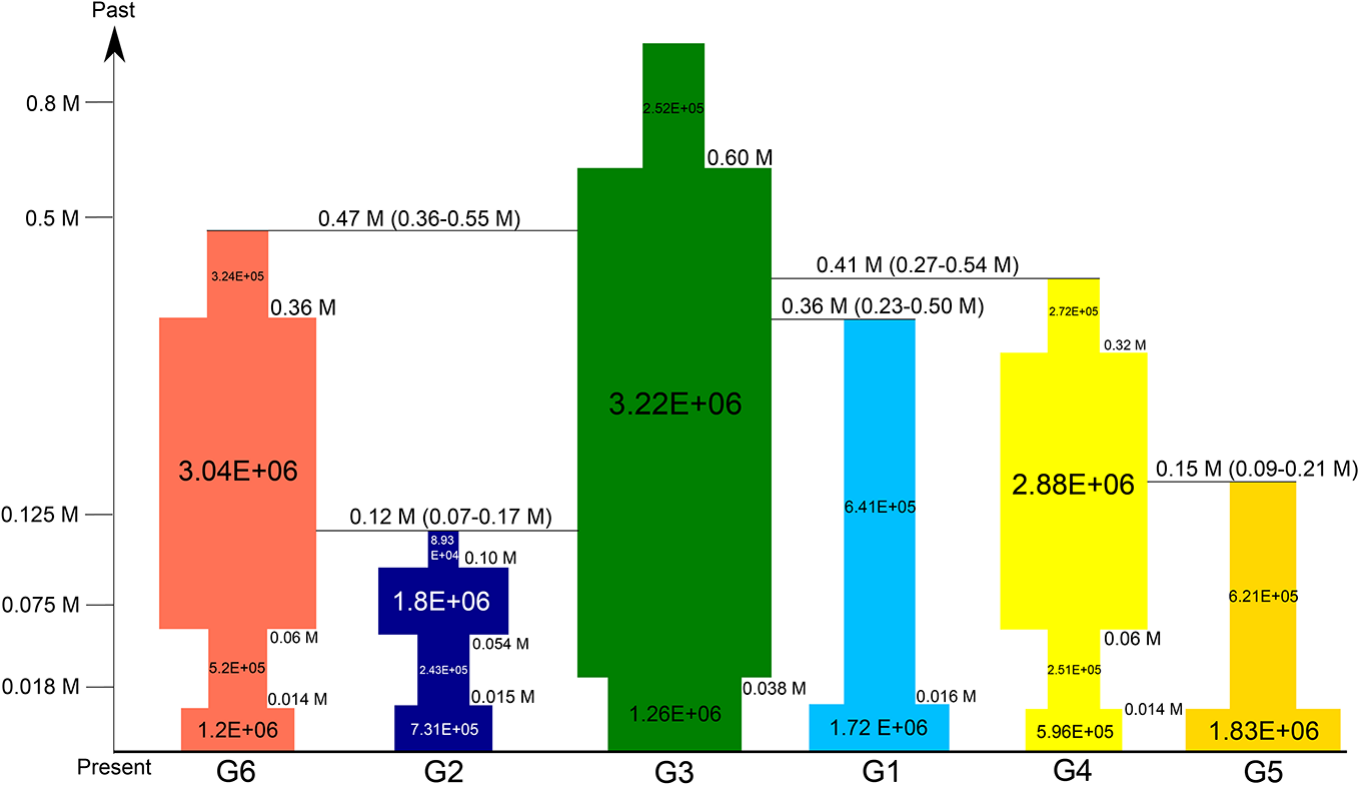
Summary of inferred demographic history of the six groups of *P. fasciculata*. Changes in population sizes are integrated into the divergent scenario. Times on the vertical axis represent the glaciation periods that occurred in the QTP (Zheng et al., 2002). Population sizes are indicated on each square. Times of divergence and changes in population sizes are indicated next to each change in population size. Only the mean values are shown (see **Tables S8** and **S9** for 95% credible interval for all values).

We estimated the divergence times and the population sizes as well as the timing and extent of these changes for the six groups. Group G3 was found to be the ancestor of *P. fasciculata* and started to expand its distribution ca. 0.60 Ma (95% highest posterior density (HPD): 0.27–0.86 Ma; **Figures 6** and **S3**; **Table S8**), followed by a slight bottleneck around 0.038 Ma (HPD: 0.004–0.075 Ma). G6 diverged from the ancestral populations formed by G3 ca. 0.47 Ma (HPD: 0.38–0.55 Ma; **Table S9**). It started to expand until ca. 0.36 Ma (HPD: 0.18–0.49 Ma), before experiencing a bottleneck ca. 0.06 Ma (HPD: 0.02–0.09 Ma; **Table S8**). Then, it quickly expanded just after the LGM. During the first expansion of this group, it came into secondary contact with the ancestral populations of G3, exchanged genes and resulted in the formation of G2 around 0.12 Ma (HPD: 0.07–0.17 Ma; **Table S9**). G2 experienced ancient expansion (0.10 Ma) and shrinkage (0.054 Ma) before and during the last glaciation (i.e., 0.015–0.075 Ma), respectively, and a recent expansion after the LGM. G1 diverged from the ancestral populations ca. 0.36 Ma (HPD: 0.23–0.49 Ma) and stayed stable through time before experiencing a recent expansion after the LGM. G4 diverged from G3 ca. 0.41 Ma (HPD: 0.26–0.54 Ma) and started to expand before experiencing a bottleneck during the last glaciation. A recent expansion after the LGM was also detected for this group. G5 was isolated from the ancient expansion of G4 (0.15 Ma, HPD: 0.09–0.21 Ma; **Table S9**) and experienced a recent expansion after the LGM.

### Species Distribution Models

The value AUC obtained for the final consensus model was 0.995, therefore it is an accurate model. The predicted current potential distribution fitted very well with the actual distribution of the species. The predictions to the LGM condition suggested that *P. fasciculata* has retreated to eastern Hengduan Mountains to occupy a large region and some restricted refugia in the eastern Himalayas, while during the MIS19 (a much colder period of Quaternary), the predicted suitable habitat for the species was enormously reduced to a small region in the eastern Hengduan Mountains.

## Discussion

Based on population genomic data, we found a striking population genetic structure for *P. fasciculata* in the highly fragmented biodiversity hotspot of the Hengduan Mountains. The patterns of genetic differentiation detected by different structure analyses were congruent, and we identified six groups of populations that capture the main characteristics of the population history of this species. The results of ABC modeling provided strong support for population divergence driven by Quaternary climatic fluctuations. The comparison of the different demographic scenarios shows that all six groups have experienced bottlenecks or stayed stable during the last glaciation, while five groups started to expand just after the LGM. These results obtained with genomic data were also supported by the SDM analyses. Taking together with a recent study that investigated factors in driving genomic variation in this species (Ren et al., 2018), our results suggest that all the historical factors (i.e., past climatic changes), spatial and environmental variables act as drivers of spatial genetic patterns. This study thus contributes a significant advance to our understanding of how alpine species were genetically structured and responded to Quaternary climatic fluctuations in the Hengduan Mountains and the QTP.

### Spatial Patterns of Genomic Diversity

Our results revealed exceptionally high levels of population divergence across the distribution of *P. fasciculata*, with a mean *F*_ST_ value of 0.377 (**Table S5**). This value is slightly lower than the level of genetic differentiation among populations reported for its closely related species *P. tibetica* (0.450; Ren et al., 2018) and *Bulbophyllum occultum* (0.387; Jaros et al., 2016), but it is still within the range usually ascribed for plants with particularly restricted dispersal ability. The divergence of populations detected with our neutral genomic markers is thus generally considered “very high” and translates into <1 migrant per generation under equilibrium conditions (Conner and Hartl, 2004), a value often considered the minimum for maintaining species cohesion. By contrast, for the species that are characterized by extensive long-distance gene flow facilitated by the dust-like and wind-dispersed pollen and seeds, the level of population differentiation usually exhibits low genetic differentiation among populations (e.g., orchids in general, *F*_ST_ = 0.02 – 0.116, Tremblay et al., 2005; epiphytic species, mean *F*_ST_ = 0.146, Phillips et al., 2012; *Restio capensis*, *F*_ST_ (RADseq/neutral) = 0.03, Lexer et al., 2014).

Spatial patterns of nuclear genomic differentiation inferred from STRUCTURE, PCA and BARRIER analyses were largely concordant with each other (**Figures 3**, **4**, and **5B**), which suggest a strong correspondence between population differentiation and their geographic locations. The pattern was further supported by the procrustes analysis, which showed a high similarity score between the overall rotated genetic space and their geographic locations (**Figure 5A**). The persistence of population divergence may be facilitated by the poor dispersal ability of the species (Richards, 2003) and reinforced by the rugged topographic features and profound ecological heterogeneity found in the Hengduan Mountains. Indeed, a recent study has shown that both spatial (i.e., geographic distance and elevation differences between populations) and environmental (i.e., climatic and edaphic variables) factors acted as drivers of population differentiation not only in selected but also in neutral genomic regions (Ren et al., 2018). Such strong correlation may suggest local adaptation, which may have further reinforced the genetic structure (Savolainen et al., 2013; Twyford et al., 2015). Furthermore, historical factors (i.e., past climatic fluctuations) were inferred to drive large-scale spatial genetic structure in this species (see below). Similar spatial, environmental, and historical factors have been suggested to drive spatial genetic patterns in its closely related species *P. tibetica* (Ren et al., 2018) and in a montane pollination-generalist herb (Muñoz-Pajares et al., 2017). By contrast, for *P. nutans*, another closely related species of *P. fasciculata*, geographic isolation played an important role in driving population divergence (Ren et al., 2018), while for the plant species *Restio capensis* that occurs in the Cape Floristic Region of South Africa, another biodiversity hotspot in the world, climatic variables were the major drivers of population divergence (Lexer et al., 2014). Therefore, drivers of population differentiation may be different and complex in different taxa and areas, and more factors should be considered when evaluating population differentiation of organisms and in particular for those that are distributed in mountainous areas.

### Demographic History of *P. fasciculata* in Response to Quaternary Climatic Fluctuations

Quaternary climatic fluctuations had a dramatic effect on distribution patterns and phylogeographic structure of species (Comes and Kadereit, 1998; Abbott et al., 2000; Hewitt, 2004), especially for those cold-adapted species distributed in high altitude such as the QTP that are assumed to be particularly vulnerable to past climatic changes (Zheng, 1996; Yao et al., 2007). Despite much effort (reviewed in Liu et al., 2014; Favre et al., 2015), we are lacking a detailed demographic history for the species present in the Hengduan Mountains and QTP because of limited genetic information (e.g., Yang et al., 2008; Du and Wang, 2016; Wan et al., 2016; but see Li et al., 2013; Shang et al., 2015). In this study, our analysis uncovers a detailed Quaternary demographic history of an alpine species distributed in the Hengduan Mountains and QTP. It corroborates our previous study on *P. tibetica*, which showed similar effects of the different factors in the Himalayas.

**Figure 7:**
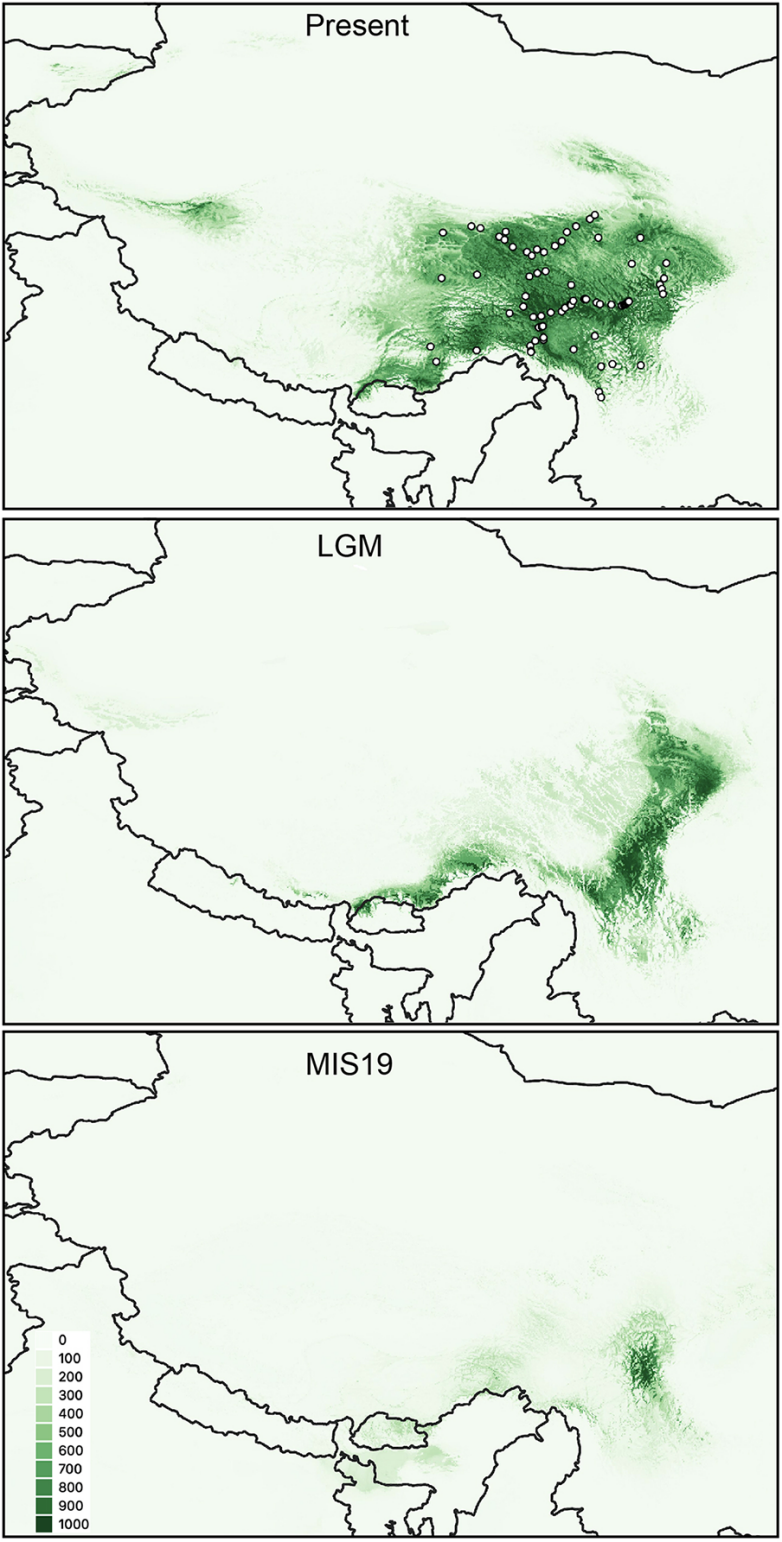
Habitat suitability of *P. fasciculata* predicted by SDMs for the present, the LGM and the MIS19. The white dots represent the 74 presences used to calibrate the models in this study.

Our test of the different demographic hypotheses done within an ABC framework shows that populations included in G3 experienced the most ancient expansions ca. 0.60 Ma (HPD: 0.27–0.86 Ma; **Figure 6**) and all other genetic groups originated from G3. The model comparisons suggest that current populations originated from ancestral populations located in the eastern Hengduan Mountains. The divergence times between the genetic groups and the ancestral populations (**Figure 6**) are dated after the largest Naynauxungla glaciation that began ca. 1.2 Ma and reached its maximum between 0.8 and 0.5 Ma in the QTP (Shi, 2002; Zheng et al., 2002). This result indicates that the Hengduan Mountains acted as a main refugium for the species to survive during this largest glaciation period, which is also indicated by the SDMs for MIS19 (**Figure 7**). The Hengduan Mountains as a refugium is also evident in the niche modeling of this species in Ren et al. (2018) and many other studies (e.g., Yang et al., 2008; Li et al., 2013; Du et al., 2017; also reviewed in Muellner-Riehl, 2019). However, a previous study suggested that *P. fasciculata* diverged from its closely related species *P. tibetica* during the Pliocene period (4.65 Ma) and expanded its distributions at the beginning of the Quaternary when the climate became cold (Ren et al., 2018). During the period between 4.65 and 0.6 Ma, it is unlikely that no population divergence had occurred, given the varied topographic features in the region. A more likely explanation would be that extensive extinction of ancestral populations might have occurred during the past environmental changes, most likely during the largest Naynauxungla glaciation, which produced an ice sheet covering an area five to seven times larger than its current range (Shi, 2002; Zheng et al., 2002; Ou et al., 2015). Such extensive ice sheet and extremely cold climate during the largest glaciation could have caused fragmentation of ancestral populations, contributing to isolation and eventual extinction of populations located at high-altitude regions, especially if one considers the fact that all the current northwestern and southwestern populations occur at more than 4,000 m (**Table S1**). By contrast, the eastern populations, occurring at lower altitude, could have survived in an eastern refugium (**Figures 6** and **7**) during the largest glaciation. When the climate became less cold, these populations could have recolonized high-altitude areas again and further gave rise to other genetic lineages triggered by the afterward glacial and interglacial events (Wang et al., 2009; Opgenoorth et al., 2010).

The timeframes of the divergence between groups G1, G4, and G6 and the ancestral populations (i.e., G3) are congruent with a period when two other glaciation events and multiple interglacial periods occurred in the QTP and Hengduan Mountains (Ou et al., 2015). The glaciations during this period became progressively less extensive, but a cold climate prevailed in the QTP until 0.17 Ma (Shi, 2002), which may have triggered these divergences. The ABC modeling of changes in populations for each group indicates that both G4 and G6 have experienced ancient expansions while G1 has stayed stable through time until the end of the LGM (**Figure 6**). Such different demographic changes may depend on their specific ecological niches (e.g., Ren et al., 2017). The current population of G1 occurs at an altitude of 4845 m. The cold climate and fewer available ecological niches as indicated in the SDMs for this population (**Figure 7**) may have prevented the ancient expansion of this group. By contrast, the current populations of the other two groups G4 and G6 occur at lower altitudes (4,170 m and average 4,583 m, respectively). The open new habitats may have facilitated their ancient expansions.

Finally, the remaining two groups G2 and G5 were formed in different ways during the last interglacial when the climate was warm (**Figure 6**). It seems that during the ancient expansions of G6 and ancestral populations (G3), the two groups came into secondary contact and resulted in the formation of G2. The divergence between G5 and G4 may due to complex topographic features in this region (**Figure 1**). The deep valleys and high mountains may have caused fragmentation of the ancient expansion of G4, reduced gene flow between them and reinforced divergence. Taken together, Quaternary climatic fluctuations pre-dating the LGM have had a much stronger influence on the evolutionary histories of plants in the QTP and Hengduan Mountains than previously thought (Qiu et al., 2011; Li et al., 2013; Muellner-Riehl, 2019), especially the largest glaciation period which may have caused massive extinction of ancient populations of plants (see also Ren et al., 2017). However, the results of this study, combined with previous studies (Wang et al., 2009; Opgenoorth et al., 2010; Ren et al., 2017), clearly indicate that the alpine species in the QTP and its adjacent regions could have survived in different refugia at high altitude, conflicting with Renner's opinion that a unique ice-sheet had covered the QTP (Kuhle, 1998; Renner, 2016). Recently, Muellner-Riehl (2019) has provided a nice glacial map in his **Figure 4**, which provides the currently most commonly accepted scheme of glaciation in the region. Furthermore, all genetic lineages have experienced bottlenecks or remained stable during the last glaciation and post-glacial expansions. This result, taken together with those recently reported for other alpine herbs (Hu et al., 2016; Wan et al., 2016; Ren et al., 2017), suggests that alpine plant species survived the last glaciation (i.e., 0.015–0.075 Ma) in multiple refugia in the QTP where most of the diverged lineages were preserved.

It should be noted that the timeframes of divergence estimated by DIY-ABC were converted into years using a generation time of one year. Whereas generation times for perennial species could be different and the biological characteristics of the species are not well described, our results using one year per generation are therefore should be treated with caution. However, another study on related species of *Primula* (Yan et al., 2012) has also used one year to study the demography history of *P. obconica*, and our ABC results are well consistent with the SDMs under past climatic conditions, which may suggest that a generation time of one year for *P. fasciculata* is acceptable. Further field and experimental studies are needed to confirm this assumption.

## Conclusions

Our analysis of population genomic data in a spatially and ecologically explicit context using appropriate model comparisons could identify the genetic structure and test several hypotheses about the detailed demographic history of an alpine plant species. Our model-testing framework combined niche modeling allowed us to demonstrate a clear effect of past climatic changes on the intraspecific divergence of *P. fasciculata*. Knowing these possible effects of past climatic changes on current populations may be useful for predicting their future range dynamics in facing ongoing climatic warming and for future management strategies. Although the 12 populations (17–20 individuals were selected from each population) are selected from our 54 sampled populations based on geographical distributions and ecological variables, bias on the genetic grouping and inferences regarding populations size and dating may exist because of the relatively small sampling size. More populations, especially the sampling sites with limited dispersal area are needed to further uncover its evolutionary history. Nevertheless, our results on *P. fasciculata*, considered in light of results recently reported for its closely related, Himalayan species *P. tibetic*a (Ren et al., 2017), and a study that investigated interspecific divergence between them (Ren et al., 2018), suggest that the largest glaciation has markedly affected the evolution and demography of these two species. Additionally, it probably caused extensive extinction of their ancestral populations. The ancestors of the current divergence populations may have survived in the Hengduan Mountains refugium or microrefugia in the Himalayas (Xing and Ree, 2017; Muellner-Riehl, 2019). Subsequent episodes of divergence are associated with following climatic fluctuations. By contrast, the LGM had less effect on recently diverged lineages that may have survived in multiple refugia, as also suggested by other studies in this area (e.g., Wang et al., 2009; Opgenoorth et al., 2010; Li et al., 2013; Hu et al., 2016) and in other mountains (e.g., European mountains, Theodoridis et al., 2016; North America, Beatty and Provan, 2010; Anatolian mountains, Ansell et al., 2011; South America, Cosacov et al., 2010; Turchetto-Zolet et al., 2013). This response pattern to past climatic changes may be also applicable for other alpine plant species in the QTP that share a preference for cold environments.

## Data Availability Statement

The dataset of *P. fasciculata* (Dryad doi: https://doi.org/10.5061/dryad.tt8n46q; File 1-D2) used in Ren et al. (2018) that comprises 234 individuals from 12 populations was re-analyzed in this study.

## Author Contributions

GR and NS planned and designed the research. GR carried out the sampling and the laboratory work, performed the molecular analysis. RM performed the SDM analysis. GR and NS wrote the manuscript with the help of EC.

## Conflict of Interest

The authors declare that the research was conducted in the absence of any commercial or financial relationships that could be construed as a potential conflict of interest.
