# Supplementary material for: Population Genetic Structure and Demographic History of Primula fasciculata in Southwest China
Source: Front Plant Sci. 2020 Jul 2;11:986. doi: 10.3389/fpls.2020.00986 (PMC7351516; doi:10.3389/fpls.2020.00986)
Supplement: Supplementary file 1 [file DataSheet_1.docx]

**Table S1** Locations of 12 populations of *P. fasciculata.* N, number of individuals used for RAD sequencing from each population.

| Population | Location | N | Longitude (°) | Latitude (°) | Altitude (m) |
| --- | --- | --- | --- | --- | --- |
| PF01 | Hongyuan, Sichuan, China | 20 | 102.365 | 32.518 | 3534 |
| PF02 | Zongke, Sichuan, China | 20 | 100.994 | 31.773 | 3521 |
| PF03 | Xinluhai, Sichuan, China | 20 | 99.058 | 31.879 | 4151 |
| PF04 | Shiqu, Sichuan, China | 17 | 98.384 | 32.543 | 4212 |
| PF05 | Changdu, Tibet, China | 20 | 97.253 | 30.692 | 4170 |
| PF06 | Ranwu, Tibet, China | 20 | 96.699 | 29.838 | 4009 |
| PF07 | Riduo, Tibet, China | 19 | 92.348 | 29.803 | 4845 |
| PF08 | Maduo, Qinghai, China | 19 | 98.703 | 35.066 | 4476 |
| PF09 | Qingshuihe, Qinghai, China | 20 | 97.302 | 33.934 | 4492 |
| PF10 | Yushu, Qinghai, China | 20 | 96.6558 | 32.874 | 4379 |
| PF11 | Qumalai, Qinghai, China | 20 | 95.939 | 34.161 | 4807 |
| PF12 | Budongquan, Qinghai, China | 19 | 94.131 | 35.068 | 4476 |

**Table S2** Information of populations assigned to different lineages or groups based on five grouping strategies that are used for AMOVAs or ABC analysis.

| Grouping strategy | L1/G1 | L2/G2 | L3/G3 | G4 | G5 | G6 | G7 |
| --- | --- | --- | --- | --- | --- | --- | --- |
| Three lineages | PF05, PF06 | PF09-PF12 | PF01-PF04, PF07, PF08 |  |  |  |  |
| Four groups | PF01-PF04, PF07 | PF05, PF06 | PF08 | PF09-PF12 |  |  |  |
| Five groups | PF01-PF04 | PF05, PF06 | PF07 | PF08 | PF09-PF12 |  |  |
| **Six groups** | **PF07** | **PF08** | **PF01-PF04** | **PF05** | **PF06** | **PF09-PF12** |  |
| Seven groups | PF01, PF04 | PF02, PF03 | PF05 | PF06 | PF07 | PF08 | PF09-PF12 |

**Table S3** Descriptions of prior settings for all parameters used in the three-steps DIY-ABC. Population size parameters are in units of population effective size, while time parameters are in units of generations.

| Parameters | Prior distributions | | | | | |
| --- | --- | --- | --- | --- | --- | --- |
| Step 1 | **Three lineages (3919 SNPs)** | |  |  |  |  |
| NL1 | Uniform[1E4-1E6] |  | t1 | Uniform[1E1-5E5] |  |  |
| NL2 | Uniform[1E1-5E5] |  | t2 | Uniform[1E1-1E6] |  |  |
| NL3 | Uniform[1E5-1E7] |  | t3 | Uniform[1E1-1E6] |  |  |
| NA | Uniform[1E1-2E5] |  |  |  |  |  |
| Step 2 | **Six groups (3919 SNPs)** | |  |  |  |  |
| N1 | Uniform[1E4-3E5] |  | t1 | Uniform[1E1-2E5] |  |  |
| N2 | Uniform[1E4-1E6] |  | t2 | Uniform[1E1-4E5] |  |  |
| N3 | Uniform[1E4-5E6] |  | t3 | Uniform[1E1-6E5] |  |  |
| N4 | Uniform[1E4-5E6] |  | t4 | Uniform[1E1-6E5] |  |  |
| N5 | Uniform[1E4-3E5] |  | t5 | Uniform[1E1-6E5] |  |  |
| N6 | Uniform[1E4-1E6] |  | t6 | Uniform[1E1-1E6] |  |  |
| NA | Uniform[1E1-5E5] |  | r | Uniform[0.001-0.999] |  |  |
| Step 3 | **G1 (9105 SNPs)** | **G2 (2402 SNPs)** | **G3 (2968 SNPs)** | **G4 (3926 SNPs)** | **G5 (5433 SNPs)** | **G6 (9198 SNPs)** |
| N1 | Uniform[1E4-2E6] | Uniform[1E4-2E6] | Uniform[1E4-3E6] | Uniform[1E4-1E6] | Uniform[1E4-3E6] | Uniform[1E4-2E6] |
| Nb | Uniform[1E4-5E5] | Uniform[1E4-1E6] | Uniform[1E4-1E6] | Uniform[1E4-5E5] | Uniform[1E4-5E5] | Uniform[1E4-1E6] |
| Ne | Uniform[1E4-5E6] | Uniform[1E4-3E6] | Uniform[1E4-5E6] | Uniform[1E4-5E6] | Uniform[1E4-5E6] | Uniform[1E4-5E6] |
| Nanc | Uniform[1E4-5E5] | Uniform[1E4-6E5] | Uniform[1E5-1E6] | Uniform[1E4-1E6] | Uniform[1E4-1E6] | Uniform[1E4-1E6] |
| t1 | Uniform[1E1-8E4] | Uniform[1E1-5E4] | Uniform[1E1-4E4] | Uniform[1E1-3E4] | Uniform[1E1-3E4] | Uniform[1E1-3E4] |
| tb | Uniform[1E1-5E5] | Uniform[1E1-1E5] | Uniform[1E1-3E5] | Uniform[1E1-1E5] | Uniform[1E1-6E4] | Uniform[1E1-1E5] |
| t2 | Uniform[1E1-9E5] | Uniform[1E1-13E4] | Uniform[1E1-5E5] | Uniform[1E1-4.2E5] | Uniform[1E1-1E5] | Uniform[1E1-5E5] |

**Table S4** AMOVAs for neutral genomic variation based on several groupings of *P. fasciculata* populations.

| Grouping/source of variation | df | Variance components | Percentage of total variance | *F*-statistic^a^ |
| --- | --- | --- | --- | --- |
| *Total* |  |  |  |  |
| Within populations | 222 | 385.925 | 69.4 | -- |
| Among populations | 11 | 170.004 | 30.6 | *F*_ST_=0.306 |
|  |  |  |  |  |
| *Three lineages* |  |  |  |  |
| Within populations | 222 | 385.246 | 65.7 | *F*_ST_=0.343 |
| Among populations | 9 | 108.798 | 18.6 | *F*_SC_=0.220 |
| Among lineages | 2 | 91.935 | 15.7 | *F*_CT_=0.157 |
|  |  |  |  |  |
| *Four groups* |  |  |  |  |
| Within populations | 222 | 385.862 | 66.8 | *F*_ST_ =0.332 |
| Among populations | 8 | 103.728 | 18.0 | *F*_SC_ =0.212 |
| Among groups | 3 | 88.247 | 15.3 | *F*_CT_ =0.153 |
|  |  |  |  |  |
| *Five groups* |  |  |  |  |
| Within populations | 222 | 386.703 | 66.9 | *F*_ST_ =0.331 |
| Among populations | 7 | 82.003 | 14.2 | *F*_SC_ =0.175 |
| Among groups | 4 | 109.023 | 18.9 | *F*_CT_ =0.189 |
|  |  |  |  |  |
| *Six groups* |  |  |  |  |
| Within populations | 222 | 386.743 | 66.7 | *F*_ST_ =0.333 |
| Among populations | 6 | 64.083 | 11.1 | *F*_SC_ =0.142 |
| Among groups | 5 | 128.675 | 22.2 | *F*_CT_ =0.222 |
|  |  |  |  |  |
| *Seven groups* | | | | |
| Within populations | 222 | 385.383 | 67.7 | *F*_ST_ =0.323 |
| Among populations | 5 | 71.91 | 12.6 | *F*_SC_ =0.157 |
| Among groups | 6 | 111.893 | 19.7 | *F*_CT_ =0.197 |

Abbreviations: AMOVAs, analyses of molecular variance; df=degrees of freedom;

^a^ All *F*-values were significant (*P* < 0.001) based on 1000 permutations.

**Table S5** Population pairwise *F*_ST_ values. All values are significant.

|  | PF01 | PF02 | PF03 | PF04 | PF05 | PF06 | PF07 | PF08 | PF09 | PF10 | PF11 |
| --- | --- | --- | --- | --- | --- | --- | --- | --- | --- | --- | --- |
| PF02 | 0.406 |  |  |  |  |  |  |  |  |  |  |
| PF03 | 0.375 | 0.340 |  |  |  |  |  |  |  |  |  |
| PF04 | 0.375 | 0.414 | 0.345 |  |  |  |  |  |  |  |  |
| PF05 | 0.410 | 0.428 | 0.360 | 0.380 |  |  |  |  |  |  |  |
| PF06 | 0.529 | 0.553 | 0.491 | 0.515 | 0.386 |  |  |  |  |  |  |
| PF07 | 0.486 | 0.531 | 0.475 | 0.464 | 0.500 | 0.604 |  |  |  |  |  |
| PF08 | 0.281 | 0.332 | 0.275 | 0.232 | 0.331 | 0.466 | 0.399 |  |  |  |  |
| PF09 | 0.396 | 0.451 | 0.401 | 0.366 | 0.423 | 0.542 | 0.457 | 0.213 |  |  |  |
| PF10 | 0.366 | 0.414 | 0.365 | 0.341 | 0.386 | 0.515 | 0.429 | 0.220 | 0.177 |  |  |
| PF11 | 0.368 | 0.414 | 0.367 | 0.342 | 0.392 | 0.511 | 0.429 | 0.203 | 0.100 | 0.128 |  |
| PF12 | 0.380 | 0.429 | 0.380 | 0.355 | 0.404 | 0.524 | 0.440 | 0.219 | 0.123 | 0.155 | 0.088 |

**Table S6** Posterior probabilities of modeled scenarios obtained by logistic regression of 1% of the closest simulated datasets for the three steps ABC modeling. The best-fit scenarios are shown in bold.

|  | Step 1 |  | Step 2 |  | Step 3 | | | | | |
| --- | --- | --- | --- | --- | --- | --- | --- | --- | --- | --- |
| Scenarios | Three lineages | | Six groups |  | G1 | G2 | G3 | G4 | G5 | G6 |
| Scenario 1 | 0 (0-0) |  | **0.93**  **(0.93-0.94)** | | **0.74**  **(0.73-0.74)** | 0.02  (0.01-0.023) | 0.01  (0-0.014) | 0(0-0) | **0.51**  **(0.5-0.52)** | 0(0-0) |
| Scenario 2 | 0 (0-0) |  | 0.06  (0.06-0.07) | | 0.1  (0.09-0.1) | 0.23  (0.22-0.24) | 0.3  (0.29-0.30) | 0(0-0) | 0.22  (0.2-0.22) | 0.34  (0.33-0.34) |
| Scenario 3 | 0.01 (0-0.08) | |  |  | 0.08  (0.07-0.08) | 0.32  (0.31-0.33) | **0.62**  **(0.61-0.63)** | 0.23  (0.22-0.24) | 0.19  (0.18-0.19) | 0.18  (0.18-0.19) |
| Scenario 4 | 0.07 (0-0.29) | |  |  | 0.08  (0.08-0.09) | **0.43**  **(0.42-0.44)** | 0.07  (0.07-0.08) | **0.76**  **(0.76-0.77)** | 0.09  (0.08-0.09) | **0.48**  **(0.47-0.48)** |
| Scenario 5 | 0 (0-0.04) |  |  |  |  |  |  |  |  |  |
| Scenario 6 | 0 (0-0) |  |  |  |  |  |  |  |  |  |
| Scenario 7 | 0 (0-0) |  |  |  |  |  |  |  |  |  |
| Scenario 8 | 0 (0-0.61) |  |  |  |  |  |  |  |  |  |
| Scenario 9 | 0 (0-0.48) |  |  |  |  |  |  |  |  |  |
| Scenario 10 | 0 (0-0.01) |  |  |  |  |  |  |  |  |  |
| Scenario 11 | 0 (0-0.14) |  |  |  |  |  |  |  |  |  |
| Scenario 12 | 0 (0-0.34) |  |  |  |  |  |  |  |  |  |
| Scenario 13 | **0.995 (0.99-1)** | |  |  |  |  |  |  |  |  |

**Table S7** Model-checking performed in DIY-ABC for steps 1 and 2. Number of outlying statistics with different levels of significance was shown for each scenario. Note that for step 3, none of statistics (total number was 4) was outlying across all six groups. The best-supported scenario for each step was shown in bold.

|  | S^*^1 | S2 | S3 | S4 | S5 | S6 | S7 | S8 | S9 | S10 | S11 | S12 | S13 |
| --- | --- | --- | --- | --- | --- | --- | --- | --- | --- | --- | --- | --- | --- |
| **STEP 1** (total number of statistics was 36) | | | | | | | | | | | | | |
| *P* < 0.05 | 5 | 6 | 11 | 5 | 9 | 12 | 5 | 6 | 4 | 7 | 7 | 4 | **4** |
| *P* < 0.01 | 6 | 6 | 9 | 4 | 4 | 2 | 2 | 5 | 7 | 12 | 10 | 6 | **2** |
| *P* < 0.001 | 7 | 13 | 7 | 7 | 6 | 9 | 10 | 15 | 17 | 5 | 7 | 5 | **1** |
| **STEP 2** (total number of statistics was 148) | | | | | | | | | | | | | |
| *P* < 0.05 | **12** | 18 |  |  |  |  |  |  |  |  |  |  |  |
| *P* < 0.01 | **6** | 5 |  |  |  |  |  |  |  |  |  |  |  |
| *P* < 0.001 | **2** | 7 |  |  |  |  |  |  |  |  |  |  |  |

S^*^, Scenario

**Table S8** Estimations of posterior distributions of parameters revealed by DIY-ABC for the best scenarios of changes in population sizes of the six groups, respectively. Estimation was based on 1% of the closest simulated data sets and logit transformation of parameters. Descriptions of all used parameters were listed in Table S2.

| **Parameter** | **N1** | **Nb** | **Ne** | **Nanc** | **t1** | **tb** | **t2** | **N1** | **Ne** | **Nanc** | **tb** | **t2** |
| --- | --- | --- | --- | --- | --- | --- | --- | --- | --- | --- | --- | --- |
|  | Scenario 4 (G2) | | | | | | | Scenario 3 (G3) | | | | |
| **Mean** | 7.31E+05 | 2.43E+05 | 1.80E+06 | 8.93E+04 | 1.52E+04 | 5.35E+04 | 1.03E+05 | 1.26E+06 | 3.22E+06 | 2.62E+05 | 3.75E+04 | 5.99E+05 |
| **Median** | 5.86E+05 | 2.23E+05 | 1.83E+06 | 8.66E+04 | 1.15E+04 | 5.21E+04 | 1.08E+05 | 1.31E+06 | 3.34E+06 | 2.73E+05 | 3.59E+04 | 6.19E+05 |
| **Mode** | 3.75E+05 | 1.89E+05 | 1.82E+06 | 7.17E+04 | 2.15E+03 | 4.64E+04 | 1.29E+05 | 1.75E+06 | 3.30E+06 | 3.28E+05 | 1.10E+04 | 6.98E+05 |
| **95% CI** | 2.46E+05 | 6.38E+04 | 6.40E+05 | 3.19E+04 | 1.06E+03 | 1.74E+04 | 6.01E+04 | 4.19E+05 | 1.09E+06 | 1.00E+05 | 3.60E+03 | 2.68E+05 |
|  | 1.68E+06 | 4.88E+05 | 2.87E+06 | 1.56E+05 | 4.19E+04 | 9.33E+04 | 1.28E+05 | 1.92E+06 | 4.81E+06 | 3.86E+05 | 7.51E+04 | 8.65E+05 |
|  |  |  |  |  |  |  |  | **N1** | **Nanc** | **t1** |  |  |
|  | Scenario 4 (G4) | | | | | | | Scenario 1 (G1) | | | | |
| **Mean** | 5.96E+05 | 2.51E+05 | 2.88E+06 | 2.72E+05 | 1.38E+04 | 5.96E+04 | 3.22E+05 | 1.72E+06 | 6.41E+05 | 1.64E+04 |  |  |
| **Median** | 5.84E+05 | 2.49E+05 | 2.90E+06 | 2.72E+05 | 1.31E+04 | 5.99E+04 | 3.34E+05 | 1.70E+06 | 6.68E+05 | 1.48E+04 |  |  |
| **Mode** | 4.73E+05 | 2.71E+05 | 4.70E+06 | 2.98E+05 | 1.16E+03 | 6.88E+04 | 4.02E+05 | 1.38E+06 | 8.61E+05 | 9.29E+03 |  |  |
| **95% CI** | 2.61E+05 | 7.89E+04 | 9.16E+05 | 7.55E+04 | 1.26E+03 | 2.22E+04 | 1.90E+05 | 5.67E+05 | 2.17E+05 | 2.28E+03 |  |  |
|  | 9.52E+05 | 4.39E+05 | 4.79E+06 | 4.68E+05 | 2.79E+04 | 9.56E+04 | 4.12E+05 | 2.86E+06 | 9.68E+05 | 3.58E+04 |  |  |
|  | Scenario 4 (G6) | | | | | | | Scenario 1 (G5) | | | | |
| **Mean** | 1.20E+06 | 5.20E+05 | 3.04E+06 | 3.24E+05 | 1.44E+04 | 6.03E+04 | 3.59E+05 | 1.83E+06 | 6.21E+05 | 1.36E+04 |  |  |
| **Median** | 1.19E+06 | 5.26E+05 | 3.03E+06 | 3.35E+05 | 1.42E+04 | 6.16E+04 | 3.76E+05 | 1.84E+06 | 6.44E+05 | 1.28E+04 |  |  |
| **Mode** | 9.29E+05 | 5.26E+05 | 2.65E+06 | 3.86E+05 | 6.07E+03 | 7.13E+04 | 4.84E+05 | 1.62E+06 | 7.51E+05 | 1.12E+04 |  |  |
| **95% CI** | 5.32E+05 | 1.51E+05 | 1.29E+06 | 1.30E+05 | 1.34E+03 | 2.15E+04 | 1.77E+05 | 6.44E+05 | 2.00E+05 | 2.23E+03 |  |  |
|  | 1.90E+06 | 8.81E+05 | 4.80E+06 | 4.82E+05 | 2.83E+04 | 9.58E+04 | 4.89E+05 | 2.88E+06 | 9.62E+05 | 2.73E+04 |  |  |

**Table S9** Estimations of posterior distributions of parameters for step 2 revealed by DIY-ABC for the best scenario of demographic history of *P. fasciculata*. Estimation was based on 1% of the closest simulated data sets and logit transformation of parameters. Descriptions of all used parameters were listed in Table S2.

| Parameter | Mean | Median | Mode | 95% CI |  |
| --- | --- | --- | --- | --- | --- |
| N1 | 2.84E+06 | 2.81E+06 | 2.86E+06 | 1.69E+06 | 4.15E+06 |
| N2 | 5.53E+05 | 5.45E+05 | 5.30E+05 | 2.77E+05 | 8.65E+05 |
| N3 | 2.16E+05 | 2.19E+05 | 2.16E+05 | 1.56E+05 | 2.68E+05 |
| N4 | 3.36E+05 | 3.37E+05 | 3.33E+05 | 2.54E+05 | 4.13E+05 |
| N5 | 1.47E+05 | 1.45E+05 | 1.37E+05 | 8.24E+04 | 2.23E+05 |
| N6 | 3.71E+05 | 3.53E+05 | 3.30E+05 | 1.78E+05 | 6.46E+05 |
| NA | 1.37E+05 | 1.35E+05 | 1.36E+05 | 7.99E+04 | 1.96E+05 |
| t1 | 1.18E+05 | 1.19E+05 | 1.25E+05 | 6.83E+04 | 1.69E+05 |
| t2 | 1.48E+05 | 1.46E+05 | 1.38E+05 | 8.58E+04 | 2.10E+05 |
| t3 | 3.61E+05 | 3.62E+05 | 3.70E+05 | 2.32E+05 | 4.95E+05 |
| t4 | 4.05E+05 | 4.08E+05 | 4.11E+05 | 2.65E+05 | 5.39E+05 |
| t5 | 4.67E+05 | 4.75E+05 | 4.80E+05 | 3.57E+05 | 5.54E+05 |
| t6 | 8.85E+05 | 8.98E+05 | 9.18E+05 | 7.55E+05 | 9.73E+05 |
| r | 3.32E-01 | 3.29E-01 | 3.33E-01 | 1.46E-01 | 5.15E-01 |

**Fig. S1** The dispersion in environmental space of the position of 12 sampled populations used in our genomic analyses (marked as the large colored dots) relative to the PC values for 54 *P. fasciculata* sampling locations.

**Fig. S2** Habitat suitability of *P. fasciculata* predicted by SDMs for the LIG.


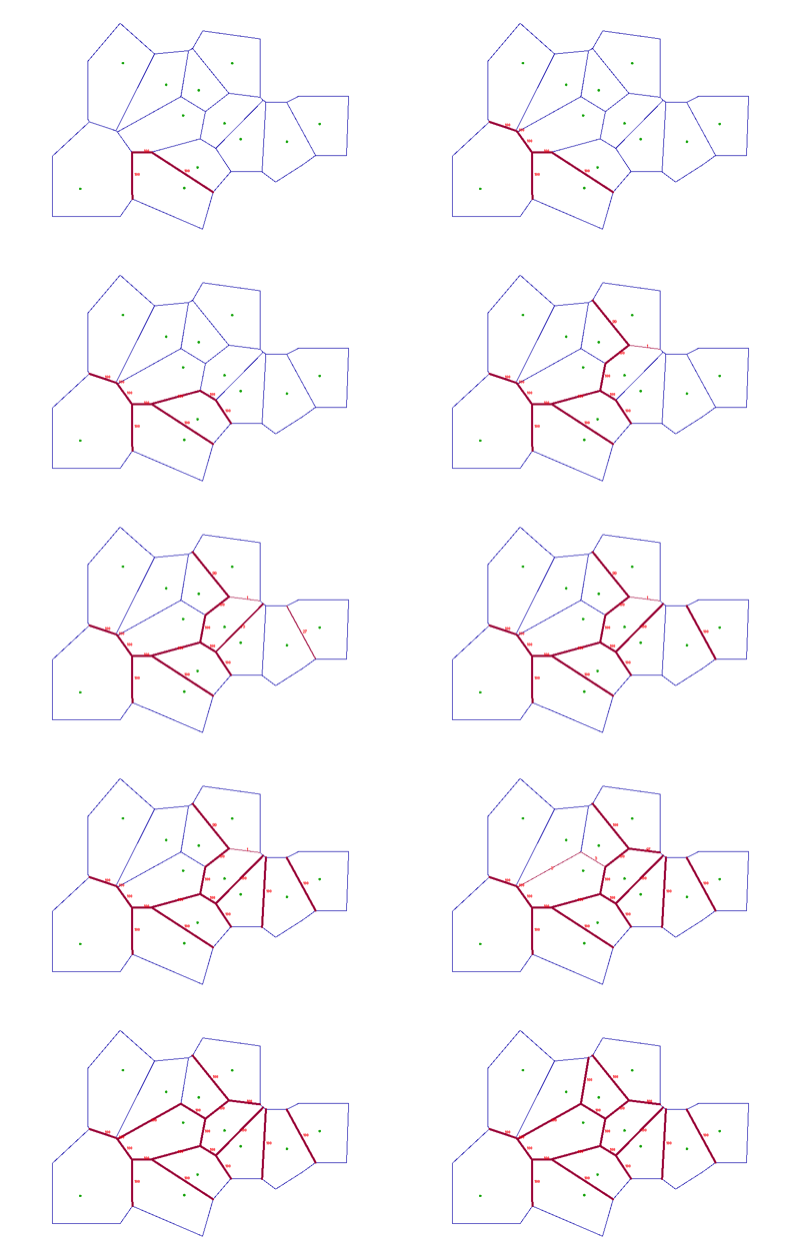


**Fig. S3** Result of BARRIER analysis shows the spatial separation of *P. fasciculata* populations for number of barriers from 1 to 10.

**Fig. S4** Plots of DIY-ABC results for three steps. (a) Plots of principal component scores along the first 2 axes (PC1, PC2) obtained from a PCA of summary statistics from the simulated demographic models (scenarios). In these PCAs, small dots with a particular color correspond to a dataset simulated with parameters drawn from the prior distributions. The yellow dot corresponds to the real dataset. (b) Posterior probabilities of competing scenarios for each step. (c) Estimations of the prior and posterior distributions of parameters revealed by DIY-ABC modeling of the best scenario for steps 1 and 2.
